# The Expression Profiles of mRNAs and lncRNAs in Buffalo Muscle Stem Cells Driving Myogenic Differentiation

**DOI:** 10.3389/fgene.2021.643497

**Published:** 2021-07-07

**Authors:** Ruimen Zhang, Jinling Wang, Zhengzhong Xiao, Chaoxia Zou, Qiang An, Hui Li, Xiaoqing Zhou, Zhuyue Wu, Deshun Shi, Yanfei Deng, Sufang Yang, Yingming Wei

**Affiliations:** ^1^State Key Laboratory for Conservation and Utilization of Subtropical Agro-Bioresources, Animal Reproduction Institute, Guangxi University, Nanning, China; ^2^The Animal Husbandry Research Institute of Guangxi Autonomous, Nanning, China; ^3^International Zhuang Medical Hospital Affiliated to Guangxi University Chinese Medicine, Nanning, China

**Keywords:** buffalo, muscle stem cells, mRNAs, non-coding RNAs, myogenesis

## Abstract

Buffalo breeding has become an important branch of the beef cattle industry. Hence, it is of great significance to study buffalo meat production and meat quality. However, the expression profiles of mRNA and long non-coding RNAs (lncRNA) molecules in muscle stem cells (MuSCs) development in buffalo have not been explored fully. We, therefore, performed mRNA and lncRNA expression profiling analysis during the proliferation and differentiation phases of MuSCs in buffalo. The results showed that there were 4,820 differentially expressed genes as well as 12,227 mRNAs and 1,352 lncRNAs. These genes were shown to be enriched in essential biological processes such as cell cycle, p53 signaling pathway, RNA transport and calcium signaling pathway. We also identified a number of functionally important genes, such as *MCMC4, SERDINE1, ISLR*, LOC102394806, and LOC102403551, and found that interference with *MYLPF* expression significantly inhibited the differentiation of MuSCs. In conclusion, our research revealed the characteristics of mRNA and lncRNA expression during the differentiation of buffalo MuSCs. This study can be used as an important reference for the study of RNA regulation during muscle development in buffalo.

## Introduction

There is an annual increase in the global consumption of beef and it is an indispensable food in our modern society, and therefore the beef cattle industry occupies an increasingly important position in modern agricultural practices (Bonny et al., 2015). According to statistics, in 2019, China's beef production was 6.85 million tons and beef imports were 1.66 million tons with a year-on-year increase of approximately 57%. It is anticipated that China's future beef demand will continue to rise. Therefore, China urgently needs a viable and thriving beef cattle industry in order to provide its society with larger amounts of high-quality beef (Mwangi et al., 2019; Ornaghi et al., 2020). There is a need for us to conduct research on the growth and meat quality of locally produced beef as well as to explore the potential molecular information of breeding stocks so as to provide reference values for future breeding protocols (Grigoletto et al., 2020).

In ruminants, skeletal muscle tissue accounts for about 40–60% of the adult animal body weight, which not only determines the level of meat production performance, but also has an important impact on meat quality. There is a group of myoblasts-muscle stem cells (MuSCs), which are the source of skeletal muscle formation and regeneration, and these have the potential for differentiation and proliferation of muscle-derived stem cells (Feige and Rudnicki, 2018; Feige et al., 2018). This is also the current cell model for studying skeletal muscle development. Under certain conditions, these cells can be activated causing the MuSCs proliferate and differentiate.

One of the main challenges in the field of muscle research is to understand how the genes that are involved in specialized muscle functions at the transcriptional and post-transcriptional levels are regulated. Undoubtedly, myogenic regulatory factors (MRFs) (Hernandez-Hernandez et al., 2017), myocyte enhancer factor-2 (*MEF2*) (Taylor and Hughes, 2017), and *PAX3*/*PAX7* genes are the main genes involved in the growth and development of skeletal muscle. Initially, long non-coding RNAs (lncRNAs) were considered to be transcriptional noise but later studies showed these RNAs play an important function in many biological processes (Jae and Dimmeler, 2020). Epigenetic control and transcriptional regulation, translation, RNA metabolism, stem cell maintenance and differentiation, autophagy and apoptosis, embryonic development, and other aspects have also been shown to play important roles (Chen et al., 2020). With the discovery of a large number of important muscle regulators such as lncRNA H19 (Xu et al., 2017), Neat1 (Wang et al., 2019), lnc-133b (Jin et al., 2017), circLOM7 (Wei et al., 2017), more and more ncRNAs related to muscle development have also been widely characterized (Martone et al., 2019). At the same time, the important role of related coding RNAs, lncRNAs, and other molecules in the development of skeletal muscle in agricultural animals are gradually being explored.

So far, with the emergence of RNA structure detection technologies such as Frag-seq (Underwood et al., 2010), (ss/dsRNA)-seq, and SHAPE-seq, have allowed scientists to characterize the structure of RNAs obtained from different tissues and cell components. When these data were combined with knowledge of RNA transformation events, such as miRNA targeting, RNA modification, and the function of RNA binding proteins (RPBs), they have emphasized the importance of RNA structure during gene regulation (Li et al., 2012). Moreover, most of these studies are focused on mRNAs and ncRNAs in order to explore the biological functions of RNA structure.

As a characteristic species of southern China, the potential use of the buffalo as a meat source has gradually attracted attention. The buffalo breeding industry has become a food basket project for urban residents, but the meat production and meat quality of buffalo needs to be improved for it to be an acceptable alternative to cattle (Li et al., 2020). Previously, several breakthroughs have been made in studies of buffalo embryos, stem cells, and somatic cells, covering traits such as milk production, reproduction, and meat production. This culminated in the successful construction of the buffalo genomic DNA sequence map (Low et al., 2019). Recently our laboratory analyzed the regulatory networks of lncRNA-mRNA interactions in the muscle tissue of cattle and buffalo (Li et al., 2020).

However, when compared to cattle, buffalo muscle has the characteristics of possessing greater shear force and consisting of thicker muscle fibers. At present, the molecular mechanisms that regulate buffalo muscle fibers formation are still unclear (Huang et al., 2021). We hypothesized that there are key signaling pathway(s) which control the myogenic differentiation of MuSCs. We, therefore, analyzed the mRNA expression of MuSCs before and after myogenic differentiation through transcriptome sequencing strategies in an attempt to screen the signal pathways that may regulate muscle fiber development. Other recent studies have also shown that differential expression lncRNAs also play an important physiological function during cellular differentiation of MuSCs (Zhu et al., 2017). This study further expands the understanding of skeletal muscle biology, and provides a reference target for the genetic improvement of buffalo and the production and cultivation of meat *in vitro* and *in vivo*.

## Materials and Methods

### MuSCs Culture and Differentiation

All experiments regarding animals were performed in the State Key Laboratory for Conservation and Utilization of Subtropical Agro-bio-resources, and were conducted in accordance with its guidelines for the care and use of laboratory animals. Primary water buffalo MuSCs were isolated and cultured from fetal-derived longissimus muscle as described in Supplementary File 1, using a combination digestion method of type I collagenase and trypsin. MuSCs were cultured in high-glucose DMEM supplemented with fetal bovine serum (Hyclone, USA; 10% FBS and 20% FBS, respectively) and antibiotics [1% penicillin and streptomycin; growth medium (GM)] at 5% CO_2_, 37°C. To induce MuSCs myogenic differentiation, MuSCs were switched to a differentiation medium (DMEM, 2% horse serum; DM) when cells were almost 90% confluent for up to 4 days.

### Sample Preparation

The tissues from Chinese buffalo at embryonic stage (90 days) were collected at a local slaughterhouse in Nanning, Guangxi province. Tissue samples, including muscle, liver, heart, lung, skin, kidney, brain, stomach, and intestine, were collected and immediately frozen in liquid nitrogen. Proliferation of MuSCs was labeled as the GM samples (*n* = 3) and differentiation of these was then called the DM samples (*n* = 3). The samples were kept at −80°C until RNA was isolated.

### Total RNA Extraction

Total RNA from cells and tissues samples were extracted with TRizol reagent (Invitrogen, Carlsbad, CA, USA) in accordance with manufacturer's instructions.

### RNA-Seq and Transcriptome Data Analysis

About 3 μg RNA per sample was used as the initial material for RNA sample preparation. PolyA-Seq libraries were prepared following the described protocol at RiboBio (Guangzhou, China) in accordance with the manufacturer's instructions. The identification of mRNAs and lncRNAs was carried out with reference to RiboBio's technical methods. We have provided a detailed description of the methods and analysis in Supplementary Document 1. All data were uploaded to the GEO database.

### Analysis of Differentially Expressed Genes, mRNAs, and lncRNAs

The RPKM (expected number of Reads Per Kilobase of transcript sequence per Millions base pairs sequenced) value was used to estimate the expression levels of mRNAs and lncRNAs. Genes with a RPKM value of <1 in no <50% of samples were defined as unreliably expressed genes, while those with a RPKM value of ≥1 in more than 50% of samples were considered as reliably expressed genes. Differentially Expressed Genes DE mRNAs, and DE lncRNAs were analyzed using DESeq2, which defined them as reliably expressed genes with |log2 (Fold Change)|>1 and *Q*-values <0.05 between any two groups.

### Gene Ontology and KEGG Analysis

Gene ontology (GO; http://www.geneontology.org) and KEGG pathway (http://www.kegg.jp) were analyzed as described previously.

### Quantitative Real-Time PCR

Total RNA was extracted using TRizol reagent (Invitrogen, Carlsbad, CA, USA) according to the manufacturer's instructions. Reverse transcription was performed by using the HiScript R II One Step RT-PCR kit (Vazyme, Nanjing, China). RT-qPCR was performed with ChamQ SYBR qPCR Master Mix (Vazyme, Nanjing, China) using the 2^−Δ*ΔCt*^ method. Beta-actin was used as the internal control. All primer sequences used are listed in Supplementary File 2.

### Western Blotting

Cells were collected from different treatment groups, pelleted by centrifugation, and then lysed in RIPA buffer. Total protein was prepared and protein concentrations were determined using the Bradford method. Proteins were then separated by SDS-polyacrylamide gel electrophoresis (SDS-PAGE) and subsequently transferred to nitrocellulose membranes. These were then blocked with 5% skimmed milk powder solution for 1.5–2 h at room temperature. The membranes were then incubated overnight with primary antibodies. Anti-PCNA, anti-CDK2, and anti-β-actin were purchased from Abcam (Cambridge, MA, USA). After that, the membranes were washed with PBS-tween and incubated for 1.5 h with horseradish peroxidase-conjugated secondary antibodies (Abcam, Cambridge, MA, USA). Protein bands were detected after treatment with Super Signal West Femto reagent purchased from Thermo (Thermo Scientific, Karlsruhe, Germany).

### Vector Construction

Construction and sequencing identification of *MYLPF* interference vectors were completed by a Biological Company (GeneCopoeia, Guangzhou, China). The interference MYLPF expression vector plasmids, which were named sh-MYLPF-A, sh-MYLPF-B, sh-MYLPF-C, and the control plasmids were named NC. All primers sequences used are shown in Supplementary File 2.

### Treatment of Cells

Muscle stem cells were grown to 70% confluence and then trypsinized and plated at 5 × 10^5^ cells/well into six-well plates (Thermo Fisher Scientific, USA). They were then transfected with vectors using X-treme GENE HP DNA Transfection Reagent (Roche, Basel, Switzerland). After incubation, the MuSCs were used for the different assays outlined below. In order to induce differentiation of myoblasts, the culture medium was changed to high-glucose DM medium.

### Immunofluorescence and Microscopy

Myoblasts of MuSCs were washed three times with PBS buffer (pH 7.4), and permeabilized for 15 min in PBS containing 0.5% Triton X-100 before fixation in PBS containing 4% paraformaldehyde for 20–30 min. Immunostaining was carried out as follows: cells were incubated overnight at 4°C with primary anti-MyoD1 (1:200; Abcam) diluted in 5% bovine serum albumin. After that, cells were washed with PBS and incubated at room temperature for 3–4 h with the corresponding secondary antibody, goat anti-mouse IgG (H+L; 1:1,000; Invitrogen) diluted in PBS. DNA was visualized using 5 mg/ml DAPI staining. Finally, the prepared cells were washed four times with PBS and observed under a fluorescence microscope (Nikon).

### Statistical Analysis

The quantitative results are presented as means ± SEMs based on at least three independent experiments. Significant variance by treatments in comparison to the untreated samples was determined by one-way ANOVA performed with GraphPad Prism version 8.0 (GraphPad Software, La Jolla, CA, USA). Differences were considered significant when *P*-values were ≤ 0.05.

## Results

### Variation of Phenotypic Characteristics During Differentiation of Buffalo MuSCs

**Figure 1:**
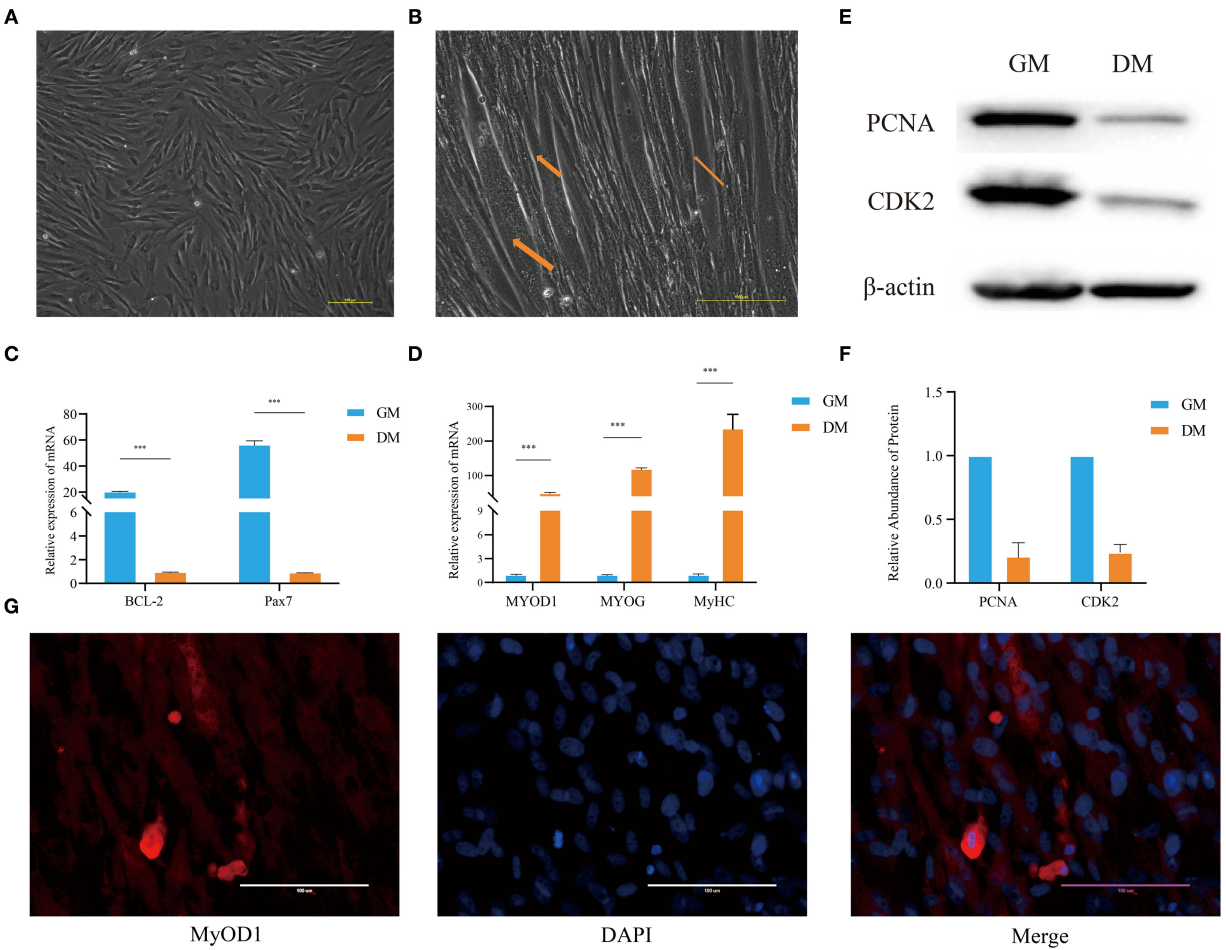
Analysis of the characteristics of buffalo MuSCs during proliferation and differentiation. **(A)** Proliferation phenotype of muscle stem cells (GM samples). **(B)** Differentiation phenotype of muscle stem cells (DM samples). **(C)** MuSCs grown as GM and DM samples were subjected to real-time PCR for *BCL-2* and *Pax7*. **(D)** The differentiation phenotype (DM) samples of MuSCs were subjected to real-time PCR for *MyoD1, MyoG*, and *MyHC*. **(E,F)** MuSCs grown as GM and DM samples were subjected to western blotting for determination of *PCNA* and *CDK2* proteins. **(G)** The differentiation phenotype (DM) samples of MuSCs were subjected to immunofluorescence for *MyOD1*. The data are presented as means ± *SD*s, *n* = 3 per group. *P < 0.05; **P < 0.01; ***P < 0.001. Scale bars = 100/200 μm.

A combination digestion method of type I collagenase and trypsin was used to obtain buffalo fetal-derived MuSCs. This cell type is similar to fibroblasts and spindle-shaped in appearance. These cells have good proliferation capacity (Figure 1A), which is referred to as the proliferation phase (GM samples) of MuSCs. In addition, when the medium was replaced with DM, after 2 days, the cells began to show myotube fusion. On the fifth day, the number of myotubes increased and the myotube fusion became more obvious, which is referred to as the differentiation phase (DM samples) of MuSCs (Figure 1B).

Western blotting showed that the expression of *PCNA* and *CDK2* in MuSCs GM samples were significantly higher than that in DM samples (Figures 1E,F). The expression levels of *BCL-2* and *Pax7* (paired Homeobox transcription factors) in GM samples were significantly higher than those in MuSCs DM samples (Figure 1C). Immunofluorescence experiments showed that the muscle marker molecule, *MyOD1*, was enriched in DM samples of MuSCs (Figure 1G). The expression levels of muscle-derived marker molecules, *MYOD1, MYOG*, and *MyHC*, increased significantly in DM samples of MuSCs (Figure 1D). These results suggest that the cells obtained were MuSCs with the capability of myogenic differentiation.

### PolyA-Seq Characteristics of Buffalo MuSCs

**Figure 2:**
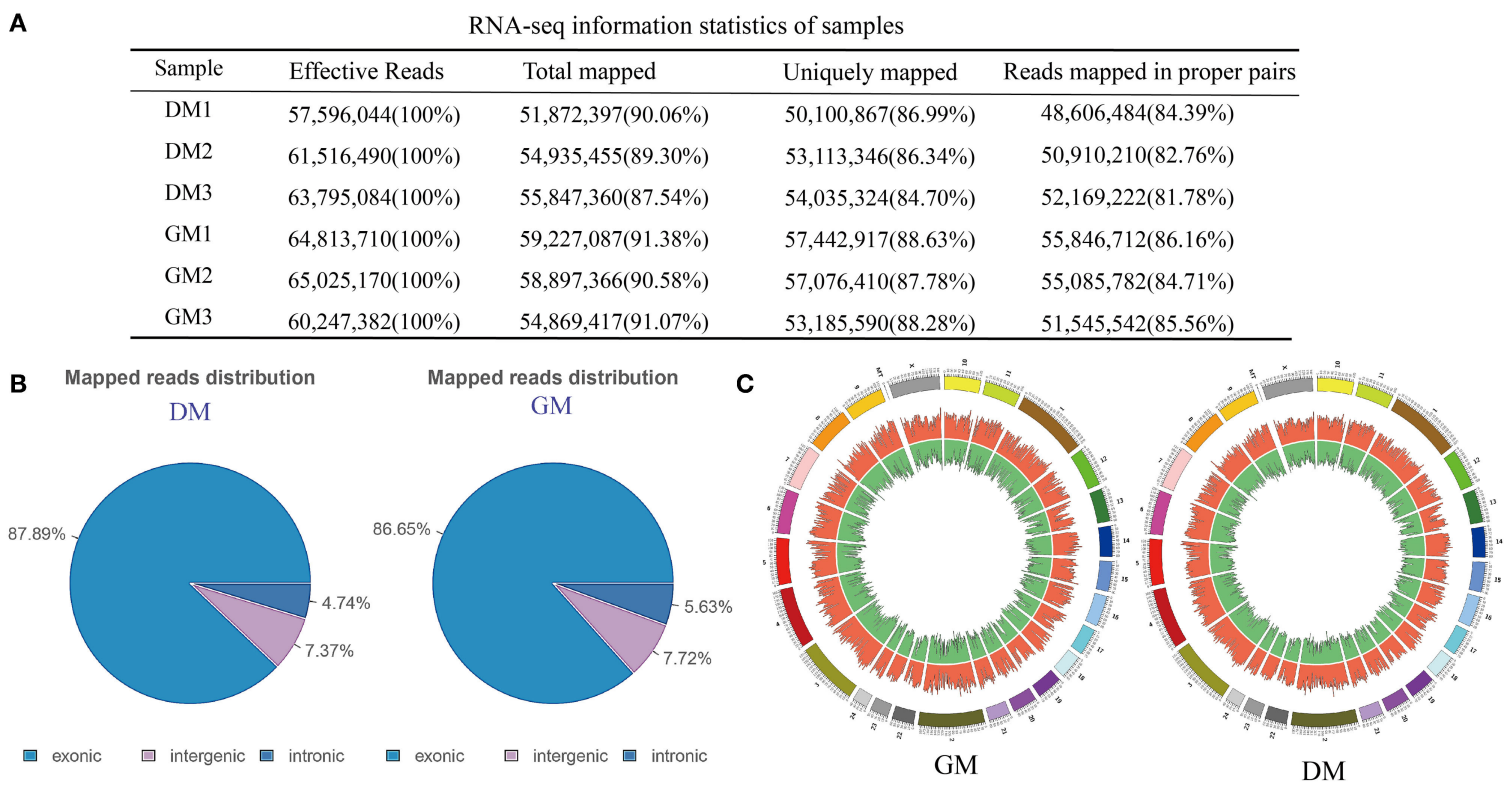
RNA-seq analysis of buffalo MuSCs during proliferation and differentiation. **(A)** RNA-seq information statistics of samples. **(B)** Mapped reads distribution. **(C)** The distribution for the reads in different chromosomes.

In order to identify the mRNAs and lncRNAs involved in proliferation and differentiation, we compared the polyA-seq status of GM and DM samples of MuSCs (Supplementary Figure S1). Analysis of sequencing data revealed that a very large number of clean reads, total maps, and uniquely mapped areas were involved in these processes (Figure 2A). The analysis of uniquely map profiles of MuSCs, showed the distribution for the reads in different chromosomes (Figures 2B,C). Among them, most of the reads from GM and DM samples were found to be targeted to exonic areas.

### Profiles of DE Genes, mRNAs, and lncRNAs During Differentiation of Buffalo MuSCs

Analysis of sequencing data revealed that a total of 31,819 genes, 57,640 mRNAs, and 11,357 lncRNAs were involved in the proliferation and differentiation of MuSCs (Supplementary Table 1). We also performed heatmaps and volcano plots for the genes, mRNAs, and lncRNAs in MuSCs (|log2 (FoldChange)|>1, *Q*-value <0.05) (Supplementary Figure S2). There were 4,820 DEGs, 12,227 DE mRNAs, and 1,352 DE lncRNAs (Supplementary Tables 2–4). Compared with the GM samples of MuSCs, 2,979 genes (61.80%), 7,505 mRNAs (61.38%), and 831 lncRNAs (61.46%) were upregulated, while 1,841 genes (38.20%), 4,722 mRNAs (38.62%), and 521 lncRNAs (38.54%) were downregulated in DM samples of MuSCs (Table 1).

**Table 1:** PolyA-seq statistics of the different results obtained.

**Differential type**	**Upregulated**	**Downregulated**	**Total**
Genes	2,979	1,841	4,820
mRNAs	7,505	4,722	12,227
lncRNAs	831	521	1,352

### Signal Pathway Enrichment Analysis of DEGs Between Proliferation and Differentiation Phases of Buffalo MuSCs

**Figure 3:**
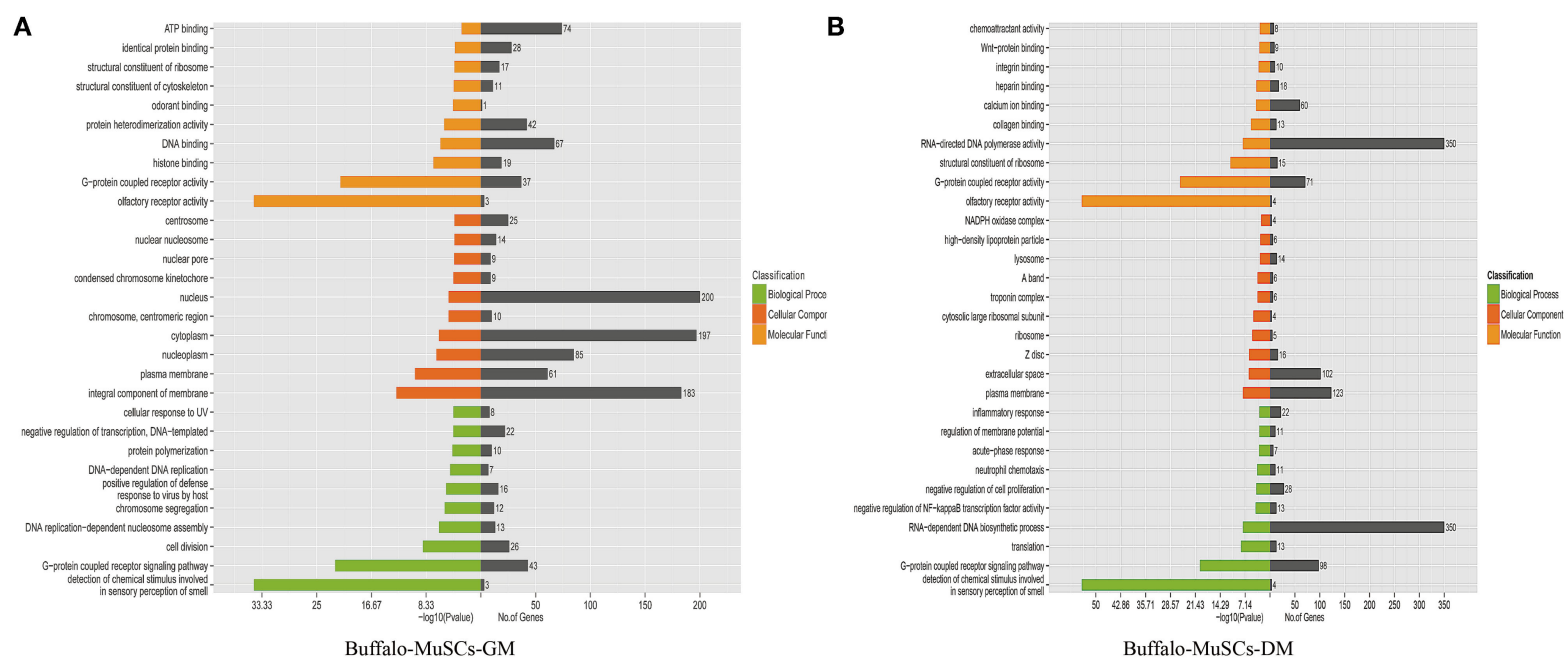
Gene ontology (GO) analysis. **(A)** GO analysis of DEGs in the GM samples of MuSCs. **(B)** GO analysis of DEGs in the DM samples of MuSCs.

**Figure 4:**
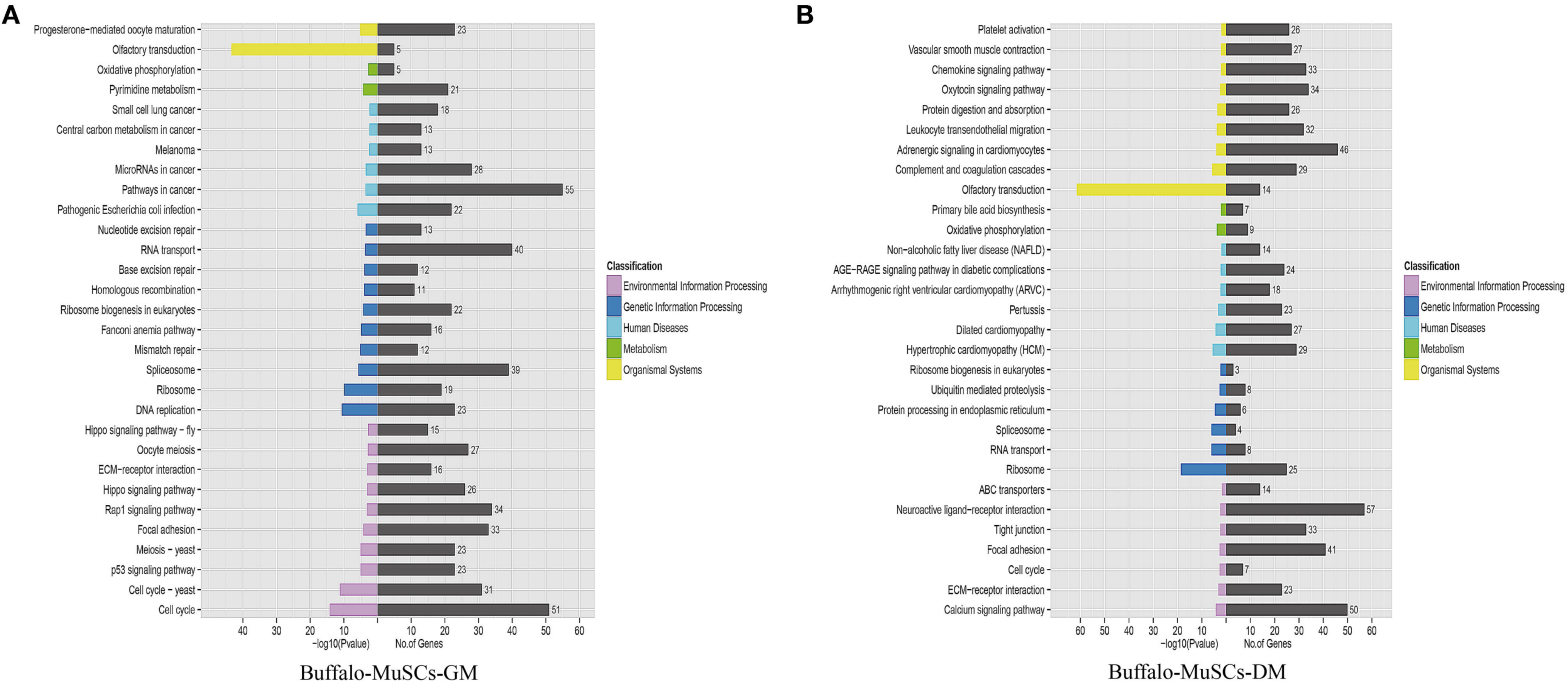
Kyoto Encyclopedia of Genes and Genomes pathway analysis. **(A)** Top 30 KEGG terms of DEGs in the GM samples of MuSCs. **(B)** Top 30 KEGG terms of DEGs in the DM samples of MuSCs.

Since we mainly analyzed mRNAs transcripts, and also involved a small number of known lncRNAs, we did not perform functional correlation analysis on these lncRNAs. We performed GO and KEGG enrichment analysis on the related regulatory DEGs in the processes of proliferation, differentiation, transformation, and maturation. This produced signal pathway information which was then used to predict the functions and mechanisms of the mRNAs (Supplementary Tables 5, 6). The results of pathway analysis of DEGs showed that GO analyzed and annotated these into three main categories: biological processes, cellular components, and molecular functions, including ATP binding and nucleus RNA-directed DNA polymerase activity (Figure 3). In addition, we employed KEGG pathway enrichment analysis to further understand the biological functions and molecular interactions of most DEGs with the assumption that the identified pathways may be involved in the development and growth of buffalo skeletal muscle. We found more than 300 pathways to be enriched, and the top 30 most significant terms were uncovered, including biological processes such as cell cycle, p53 signaling pathway, RNA transport, and calcium signaling pathway (Figure 4). In short, these signal pathways related to DEGs play an important role in the regulation of MuSC proliferation and differentiation, which provides an important basis for subsequent research on buffalo myogenesis.

### The Verification of DEGs and DE lncRNAs in MuSCs

**Figure 5:**
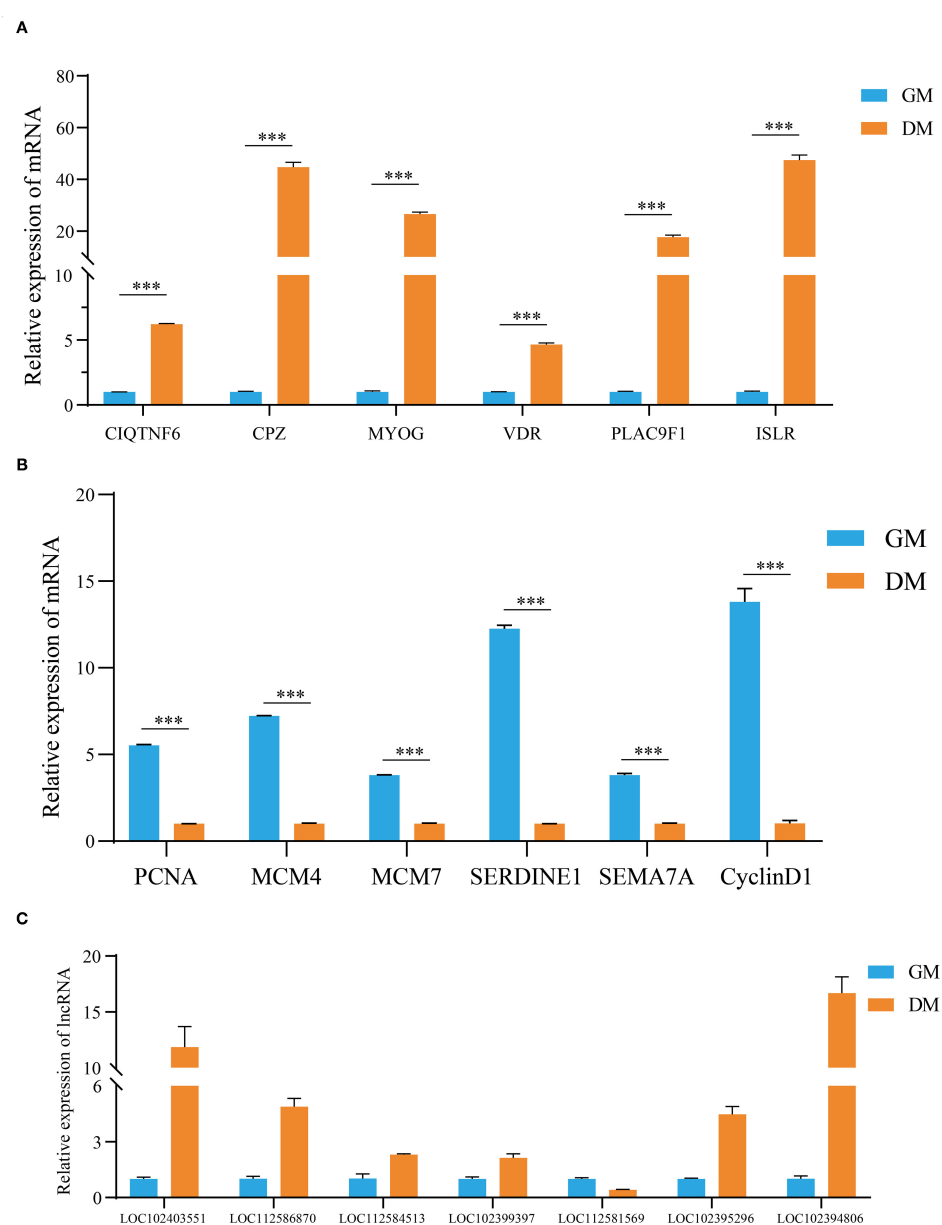
Validation of the expression levels of DEGs, DE lncRNAs by RT-qPCR. **(A,B)** The validation results of DEGs and **(C)** DE lncRNAs. The data are present as means ± SDs, *n* = 3 per group. ***P < 0.001.

Based on the expression levels of DEGs and DE lncRNAs, including 12 genes (*MCM4, MCM7, SERDINE1, SEMA7A, C1QTNF6, CPZ, VDR, PLAC9, ISLR, MyOG, PCNA*, and *cyclin D1*) related to the cell cycle, actin cytoskeleton, cell differentiation, and lipid metabolism (Figures 5A,B), and seven random lncRNAs (LOC102403551, LOC112586870, LOC112584513, LOC102399397, LOC112581569, LOC102395296, and LOC102394806; Figure 5C), were selected for RT-qPCR verification. After comparisons with the RNA-seq data, similar expression trends for RT-qPCR were discovered, showing the strong consistency between RT-qPCR and RNA-seq data.

### The Role of *MYLPF* in Buffalo MuSCs

**Figure 6:**
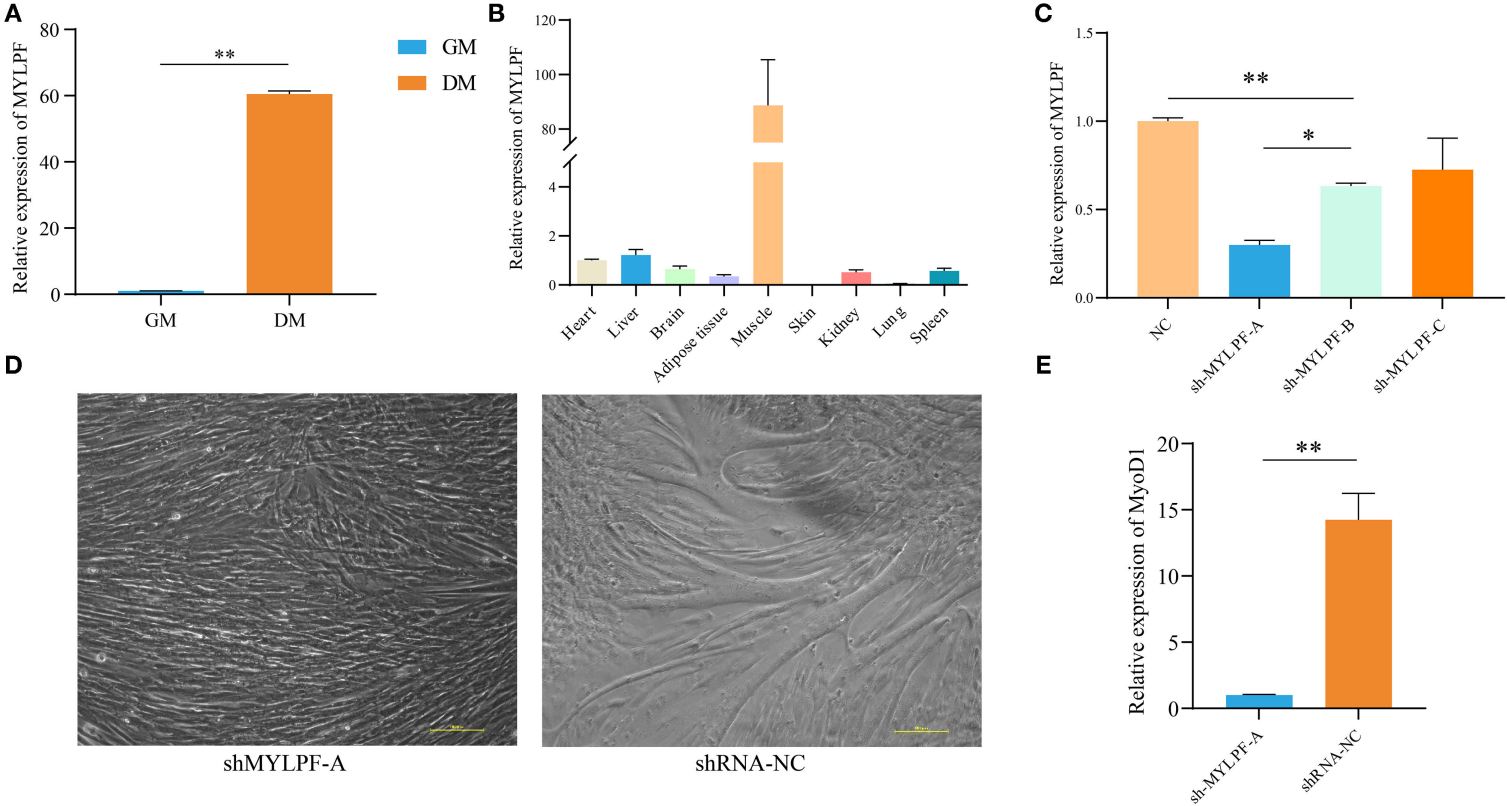
Expression and characterization of *MYLPF* in buffalo skeletal muscle. **(A)** Validation of the expression of *MYLPF* by RT-qPCR. **(B)** The expression levels of *MYLPF* in different tissues of buffalo. **(C)** The interference efficiency of *MYLPF* was measured by RT-qPCR. **(D)** Inhibition of *MYLPF* expression on myotubule formation. **(E)** The mRNA expression of myogenesis marker gene, *MyOD1*, was measured by RT-qPCR. The data are presented as means ± SDs, *n* = 3 per group. *P < 0.05; **P < 0.01. Scale bars = 100 μm.

We identified a dysregulated gene, *MYLPF*, which was shown to be upregulated significantly (by almost 60-fold) in DM compared with GM samples when measured by RT-qPCR (Figure 6A). In addition, *MYLPF* was expressed in various tissues, such as heart and liver and the highest expression levels seen in muscle (Figure 6B). A previous report also showed that the relationship between *MYLPF* and meat quality can be used as an important genetic consideration when dealing with gene-assisted selection programs. This suggests that *MYLPF* may play an important role in muscle development. Therefore, *MYLPF* was selected for more in-depth study in order to further explore its potential functions in MuSCs.

Subsequently, interference vector (sh-MYLPF-A/B/C/NC) plasmids were introduced into the 293T cell line which is derived from human embryonic kidney 293 cells and contains the SV40 T-antigen. After 24 h, the reporter gene, green fluorescence protein (GFP), was found to be expressed in the transfected cells, with a strong fluorescence observed under the fluorescence microscope (Supplementary Figure S3). Further qPCR showed that the expression levels of *MYLPF* declined by 70% in the transfected cells with the sh-MYLPF-A plasmid (Figure 6C).

Sh-MYLPF-A vectors were then transferred into P2 MuSCs (Supplementary Figure S4). The cells were cultured for a further 24 h, followed by replacement of the medium with myogenic differentiation medium. On the fourth day, the knockdown of *MYLPF* was found to have inhibited the formation of myotubes (Figure 6D). The marker gene of myoblast differentiation, *MyoD1*, was then measured by qPCR. The results showed that there were significantly lower levels of *MYLPF* in the knockdown group compared to the controls (Figure 6E). These findings suggested that *MYLPF* knockdown inhibited differentiation of MuSCs.

## Discussion

Currently, the global population is 7.7 billion, and it is expected to exceed 9 billion by 2050 (Bonny et al., 2015). By then, mankind will face a bigger challenge of food provision for the growing population, and this will have a major impact on global meat consumption which will increase accordingly (Ornaghi et al., 2020). Muscle development is an important factor that affects the growth rate, meat yield, meat quality, and other important economic traits of livestock, and this process is dependent on the proliferation and differentiation of MuSCs (Feige and Rudnicki, 2018; Boscolo Sesillo et al., 2020).

Initially, we established a successful protocol for *in vitro* culture of buffalo MuSCs, which provided a good working foundation for subsequent research on candidate factors that regulate buffalo muscle development. With the rise of *in vitro* cultured meat such as laboratory meat, the role played by MuSCs is becoming more important. The *in vitro* cultured meat production technology is still in its infancy, and it is necessary to strengthen and improve the technical systems involved in MuSCs production of beef (Bhat et al., 2017). Therefore, buffalo MuSCs can play an important role in the emerging research fields of animal husbandry, such as providing improvement of buffalo meat quality and production as well as increasing our biochemical knowledge of MuSCs *in vitro*.

In this study, we constructed the expression profiles of mRNAs and lncRNAs in the process of myogenic differentiation of buffalo MuSCs. During this process, a total of 4,820 genes, 12,227 mRNAs, and 1,352 lncRNAs were differentially expressed. Among them, 2,979 genes, 7,505 mRNAs, and 831 lncRNAs were significantly related to the myogenic differentiation of these cells, and they affected the formation of myoblasts and the fusion of myotubes. In addition, we performed target gene analysis on differentially expressed lncRNAs, and obtained many lncRNAs-target gene relationship networks. We can indirectly predict the function of the candidate lncRNA through the target gene (Supplementary Tables 7, 8). Previous studies had confirmed that compared with cattle, buffalo muscles have larger muscle fiber diameters and poorer meat texture. Of course, myotube fusion is an important factor affecting the formation of muscle fibers (Picard and Gagaoua, 2020). The mRNAs and lncRNAs which are related to myogenic differentiation of MuSCs may regulate the diameter of muscle fibers through myotube fusion, which further affected the quality of meat. We also found some key signal transduction pathways, such as p53 signal transduction pathway, TGF-β signal pathway, calcium signal transduction pathway that were related to these RNAs. These signal pathways are involved in cell development and maintenance of muscle structure and function, suggesting that they were also likely to be important regulatory signals for regulating buffalo muscle fiber hypertrophy (Liu et al., 2018; Valle-Tenney et al., 2020).

We also randomly selected a batch of candidate molecules for verification, and their expression trends were found to be consistent with the RNA-seq results, indicating that the sequencing data was reliable. We found that genes such as *VDR, PLAC9, ISLR* were involved in the myogenic differentiation process of MuSCs, but their molecular mechanisms needed to be further explored (Bass et al., 2020; Cui et al., 2020). It had been confirmed that the immunoglobulin superfamily containing leucine-rich repeats (*ISLR*) promoted skeletal muscle regeneration by activating canonical Wnt signaling. Loss of function of *ISLR* resulted in defective differentiation of myoblasts leading to a block in myotube formation (Zhang et al., 2018). Therefore, *ISLR* may be an important biological regulator to control buffalo muscle development. It had also been reported that *MYLPF* was one of the muscle-derived marker genes involved in the process of muscle metabolism and related to meat quality traits (Rosa et al., 2018; Chong et al., 2020). As one of the muscle markers, *MYLPF* is expected to become a target for regulating the quality traits of buffalo meat (Silva et al., 2019). We also found that decreased *MYLPF* was linked to an inhibition of myogenic differentiation of buffalo MuSCs, but the molecular mechanism of this phenomenon is not yet fully understood. Therefore, how *MYLPF* regulates buffalo muscle regeneration is worthy of further investigation.

At present, lncRNA is also one of the research hotspots in the field of ncRNA (Martone et al., 2019). However, we only discovered the number of known lncRNAs and their expression levels involved in the myogenic differentiation of MuSCs. Then, we screened out a batch of potential candidate lncRNAs, such as LOC102403551, LOC112586870, and LOC102394806. These potential candidate lncRNAs may affect the myoblast differentiation of MuSCs by regulating gene expression through miRNAs, RPBs, and other ways (Chi et al., 2019; Xu et al., 2019; Guo et al., 2020; Liu et al., 2020). In future studies, we and others will also investigate the interactions between lncRNAs and enhancers in order to regulate fate of MuSCs (Lin et al., 2019; Nikonova et al., 2019; Williams et al., 2020). The biological effects of these lncRNAs related to buffalo MuSCs, lncRNA evolution, lncRNA SNP issues, etc. are also worth pursuing (Qian et al., 2019). At the same time, how these lncRNAs and coding genes regulate the molecular mechanisms of farmed beef production and their contributions to the *in vitro* meat production process also need to be further explored.

In summary, we have established the mRNA and lncRNA expression profiles that regulate the myogenic differentiation of buffalo MuSCs, and further predicted and verified the signaling pathways and candidate regulators involved in cell proliferation and differentiation. These results enrich the expression information of factors that regulate the development of MuSCs in Chinese local fine beef cattle breeds, and provide effective genetic information for future programs of breeding high-yield beef cattle.

## Conclusions

In this study, the proliferation and myogenic differentiation phenotypic characteristics of buffalo MuSCs were compared for the first time, and the expression of mRNAs and lncRNAs in these cells were reviewed. Many coding RNAs and lncRNAs were found to be differentially expressed during the proliferation and myogenic differentiation phases of MuSCs. We further identified and verified a number of differentially expressed molecules such as *SERDINE1, ISLR, MYLPF*, LOC102403551, LOC112586870, and LOC112584513. This study lays the foundation for further research on the role of lncRNAs in the muscle development of buffalo with a view to improving its share as a desirable beef alternative in the marketplace.

## Data Availability Statement

The datasets presented in this study can be found in online repositories. The names of the repository/repositories and accession number(s) can be found below: NCBI GEO, accession no: GSE164808.

## Author Contributions

YD, SY, YW, JW, and RZ conceived and designed the experiments. RZ, JW, and CZ performed the experiments and analyzed the data. RZ, QA, CZ, XZ, ZW, HL, and DS contributed reagents, materials, and helped to analyze the data. RZ and ZX wrote the manuscript and SY, YD, DS, and YW revised it. All authors contributed to the article and approved the submitted version.

## Conflict of Interest

The authors declare that the research was conducted in the absence of any commercial or financial relationships that could be construed as a potential conflict of interest.

## Abbreviations

MuSCs: muscle stem cells
GO: gene ontology
KEGG: Kyoto Encyclopedia of Genes and Genomes
lncRNAs: long non-coding RNAs
RT-qPCR: quantitative real time PCR.
