# Supplementary material for: The Expression Profiles of mRNAs and lncRNAs in Buffalo Muscle Stem Cells Driving Myogenic Differentiation
Source: Front Genet. 2021 Jul 7;12:643497. doi: 10.3389/fgene.2021.643497 (PMC8294193; doi:10.3389/fgene.2021.643497)
Supplement: Supplementary file 9 [file Table_9.DOCX]

PolyA-Seq is based on eukaryotic mRNA and partial long-chain non-coding RNA (long non-coding RNA, lncRNA) with polyA tail structure at the 3 'end. RNA is captured by polyA tail and sequenced, and then the differential expression or structural variation of all genes and transcripts containing polyA structure are detected, and the molecular markers related to disease or traits are screened. PolyA-Seq can produce 6G Clean bases, which can accurately detect low abundance gene transcripts, reveal the complexity of transcripts, determine the structure of genes and transcripts, alternative splicing, RNA editing, polyA tail non-coding RNA and new transcripts.

**1．Experimental process**

The PolyA-Seq sequencing process is shown in figure 1.


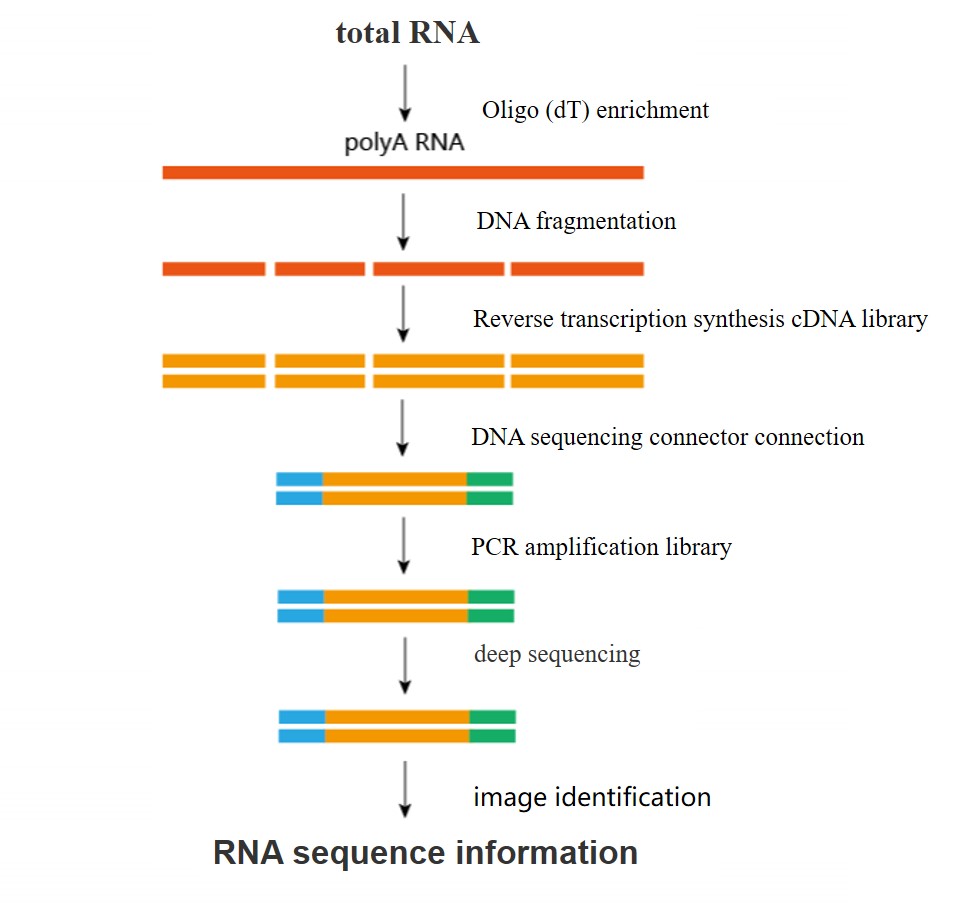


Figure 1: transcript group sequencing process

**2. Information analysis process**

After obtaining the original sequencing data (Raw Data), we first need to filter the data, disconnect the sequence and deal with the low-quality reads, and then evaluate the sequencing quality to obtain the high-quality data (Clean Data). The Clean Data was compared with the reference genome to get the BAM file.

For the detected mRNA, the gene expression was further calculated and the differentially expressed genes among samples were analyzed, and the differentially expressed genes were analyzed by Gene Ontology (GO) analysis and KEGG biological pathway enrichment analysis.

For the detected lncRNA, the expression amount of lncRNA was calculated and the differential expression lncRNA, between samples was analyzed, and specific comments were given. The specific process is shown in figure 2.


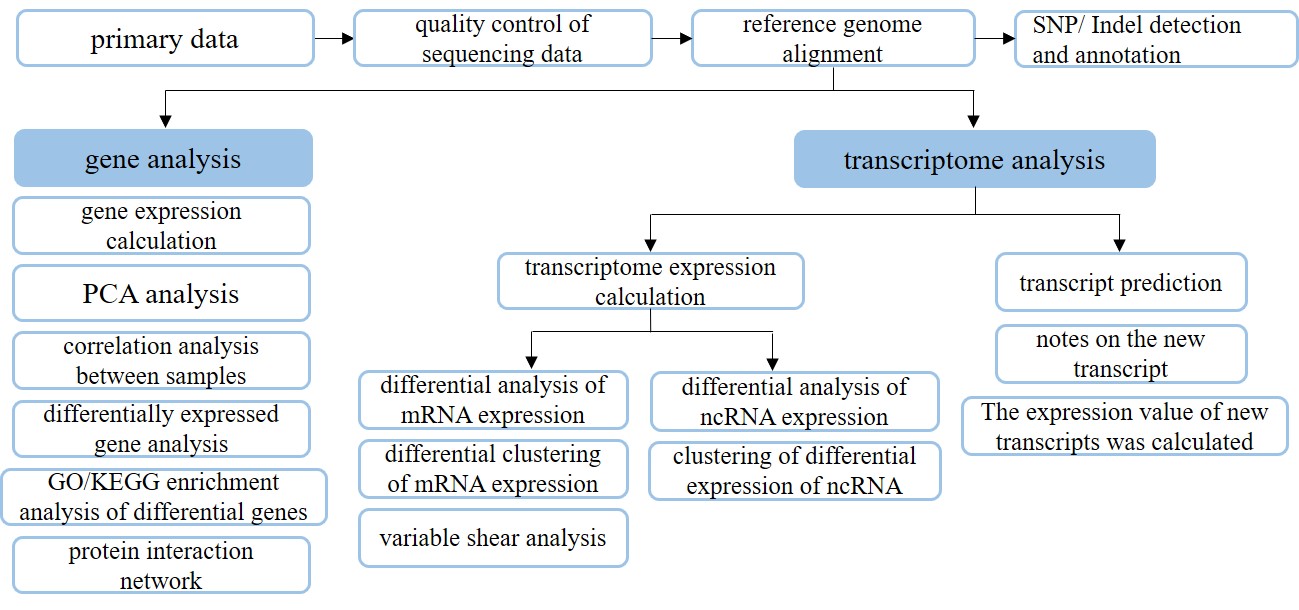


Figure 2: bioinformatics analysis process

**3. reference Genome alignment and Genome-wide reads Distribution Map**

**3.1 Reads alignment reference genome**

The link to the buffalo reference genome used in this study is as follows: https://www.ncbi.nlm.nih.gov/assembly/GCA_003121395.1.

We used HISAT2 software to compare the sequencing data with the reference genome, and makes a comprehensive evaluation on the coverage area and depth of the sequencing data. HISAT2 is the successor of TopHat2/Bowtie2, using the improved BWT algorithm (FM index), HISAT uses a large number of FM indexes to cover the whole genome, combined with several alignment strategies to achieve efficient comparison of RNA-seq data, faster speed and less resource consumption.

**3.2 data coverage**

After the alignment is completed, we count the location information of all the genomes corresponding to the reads, so as to evaluate the coverage depth of the sequencing data.

**3.3 Reads distribution results**

In the species with more complete gene annotation, the reads, on the chromosome is annotated to exonic (exon), intronic (intron) and intergenic (gene spacer region).

**3.4 Transcript coverage and uniformity results**

Under ideal conditions, the distribution of reads on transcripts should be uniform. However, due to the degradation of RNA fragments, randomness of interruptions, GC content, PCR amplification preference and other factors, the distribution of reads in transcripts will show a certain preference, which may affect the results of follow-up analysis. Therefore, in addition to counting the distribution of reads at the genome level and different gene regions, we also evaluated the uniformity of transcripts reads coverage.

**3.5 saturation evaluation of sequencing results**

In order to evaluate the adequacy of the data, the saturation of the number of genes obtained by sequencing is tested to see whether the number of newly detected genes is less and less or not with the increase of the amount of sequencing data, that is, whether the number of genes detected tends to be saturated. The Mapped Data of each sample was used to simulate the saturation of the number of genes detected, and the saturation curve was drawn as shown in figure 6 below.


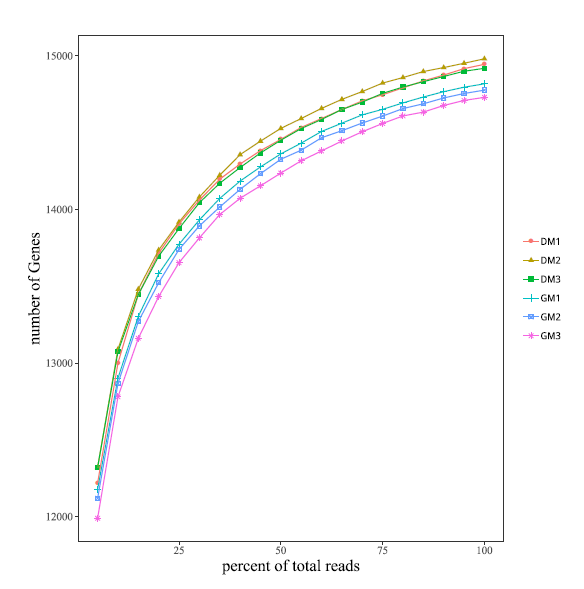


Figure 6: Sequencing saturation results of each sample

**References:**

[1] Kim D, Langmead B, Salzberg S L. HISAT: a fast spliced aligner with low memory requirements[J]. Nature methods, 2015, 12(4): 357-360.

[2] Pertea M, Kim D, Pertea G M, et al. Transcript-level expression analysis of RNA-seq experiments with HISAT, StringTie and Ballgown[J]. Nature protocols, 2016, 11(9): 1650-1667.

[3] Li H, Handsaker B, Wysoker A, Fennell T, Ruan J, et al. (2009) The sequence alignment/map format and SAM tools. Bioinformatics 25: 2078-2079.

[4] Wang K, Li M, Hakonarson H. (2010) ANNOVAR: functional annotation of genetic variants from high throughput sequencing data. Nucleic acids research 38: e164-e164.

**4.Classification of genes and transcripts**

**4.1 mRNA classification**

Messenger RNA (mRNA) is a type of single-stranded RNA that is transcribed from DNA and processed by hnRNA (heterogeneous nuclear RNA) splicing. It carries genetic information and can guide protein synthesis. Thus, mRNA is a transcript that encodes a protein.

**4.2** **Gene classification**

The genes in this study refer to molecules that have the ability to encode proteins, and contains an open reading frame (ORF). A protein coding gene that has at least one transcript with a valid ORF and one or more coding transcripts that contain a polymorphism.

**4.3 lncRNA Prediction and classification**

We used the buffalo reference genome for analysis. The annotation file is GCF_003121395.1(https://www.ncbi.nlm.nih.gov/genome/?term=Bubalus+bubalis), which belongs to parametric transcriptome sequencing and does not involve the process of genome assembly and annotation.

**#Predict the function of lncRNA**

We can predict the target genes of lncRNA through cis and trans interactions and perform GO and KEGG functional analysis of the target genes to establish a regulatory network of lncRNA-target genes-signaling pathways, and use this regulatory network to further predict the involvement of lncRNA in biological processes Features. However, we lack sufficient data to analyze the function of lncRNA, and fail to obtain information about the function of lncRNA.

**4. The overall level of gene expression**

In RNA sequencing, RPKM (expected number of Reads Per Kilobase of transcript sequence per Millions base pairs sequenced) is the number of reads from a gene per kilobase length per million reads, which takes into account both the sequencing depth and the gene. The influence of length on the count of reads is currently the most commonly used method for estimating gene expression levels. The calculation formula is as follows:


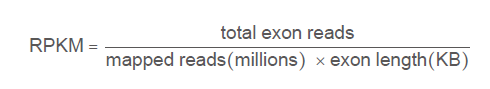


We mapped the bam file obtained by sequencing to the gtf file of the reference species to obtain the gene expression level, and performs regional statistics on the gene expression level of the genome according to the statistical method to reflect the overall expression level of the sample.


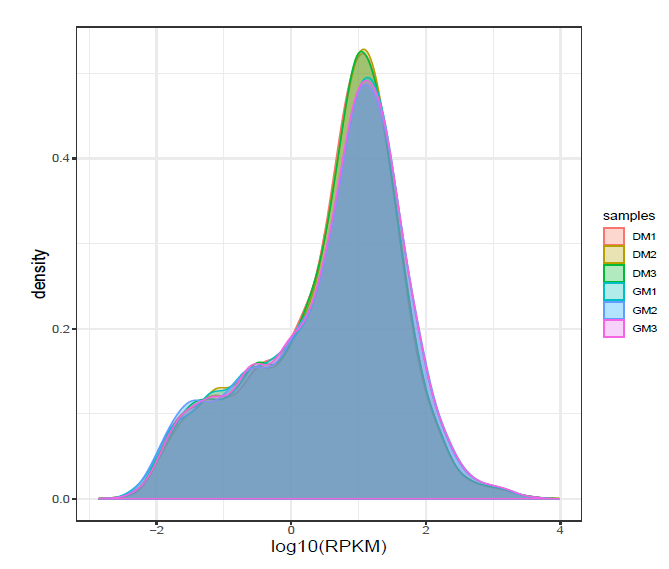


Figure 7: Gene expression density map

**References:**

[1] Mortazavi A, Williams BA, McCue K, Schaeffer L, Wold B. (2008) Mapping and quantifying mammalian transcriptomes by RNA-Seq. Nat Methods. 5(7):621-8.

[2] Anders S, Pyl P T, Huber W. HTSeq —a Python framework to work with high-throughput sequencing data[J]. Bioinformatics, 2015, 31(2): 166-169.

**5.Differentially expressed genes** **(DEGs),** **mRNAs (DE mRNAs ) and lncRNAs (DE lncRNAs) Analysis**

The differentially expressed genes (DEGs), mRNAs (DE mRNAs ) and lncRNAs (DE lncRNAs) were analyzed respectively. The gene expression difference analysis was performed using DESeq2 software, and the calculation method was based on the Negative Binomial Generalize Linear Model (Negative Binomial Generalize Linear Model). DESeq2 combines a new feature of methodology-using shrinkage estimation to calculate distribution and multiples of difference. We identified genes with a |log2(Fold Change) |>1 and a Qvalue<0.05 in a comparison as significant DEGs.

Subsequent analysis uses the DESeq2 method to perform differential analysis of mRNAs, lncRNAs. For specific methods, refer to gene expression differential analysis.

**DEGs** were then subjected to enrichment analysis of GO functions, KEGG pathways and others.

**Cluster analysis**

We used the RPKM value of the differential gene under different experimental conditions to use hierarchical clustering (Hierarchical Clustering) analysis to cluster genes with the same or similar expression patterns into clusters, and uses different colored regions to represent different clusters. Class grouping information, so as to judge the clustering mode of the regulation mode under different samples or different experimental conditions.

On the other hands, through two levels of difference multiple (|log2(FoldChange)|>1) and significance level (Qvalue<0.05), we can select the DEGs, DE mRNAs and DE lncRNAs between samples, and determine the number of DEGs, DE mRNAs and DE lncRNAs between samples and the specific significant differential expression. Gene statistics, forming biological volcano maps and cluster heat maps of DEGs, DE mRNAs and DE lncRNAs.

**References:**

[1] Anders S, Huber W. (2010) Differential expression analysis for sequence count data. Genome Biol.11(10):R106.

[2] Gao D, Kim J, Kim H, Phang TL, Selby H, Tan AC, Tong T.A survey of statistical software for analysing RNA-seq data.Hum Genomics. 2010 Oct;5(1):56-60.

[3] Benjamini Y，Hochberg Y. (1995) Controlling the False Discovery Rate: A Practical and Powerful Approach to Multiple Testing. Journal of the Royal Statistical Society. Series B (Methodological), 57(1):289-300

[4] Love M I, Huber W, Anders S. Moderated estimation of fold change and dispersion for RNA-seq data with DESeq2[J]. Genome biology, 2014, 15(12): 550.

**6.GO Enrichment Analysis**

Gene Ontology (GO) analysis can annotate the function of each gene, and calculate the most significant function of a specific series of genes through statistical analysis such as Hypergeometric Distribution.

Gene function enrichment analysis is mainly divided into two steps:

**Frist, Gene function annotation:**

The GO function annotation provides annotations for DEGs in terms of molecular function (Molecular Function), Biological Process (Biological Process) and Cellular Component (Cellular Component), thereby providing gene function classification labels and background knowledge of gene function research.

The three annotations included in GO are introduced as follows: (1) Cell composition: each part of the cell and the extracellular environment; (2) Molecular function: The main activities of gene products at the molecular level, such as binding and catalysis; (3) Biological processes : Events or behaviors that occur within the cell, which can define the beginning and the end.

**Second,** **Enrichment analysis:**

According to the GO annotations of genes, select all genes of this species as background genes, use hypergeometric distribution method to calculate the P value, and use P<0.05 as the significance threshold to obtain statistically significant high-frequency annotations relative to the background, thereby obtaining the gene set Distribution information and significance in GO categories.

**References**:

[1] A direct approach to false discovery rates. Journal of the Royal Statistical Society: Series B (Statistical Methodology), Vol. 64, No. 3. (August 2002), pp. 479-498, doi:10.1111/1467-9868.00346

[2] Gene ontology: Tool for the unification of biology. The Gene Ontology Consortium. Nature genetics, Vol.25, No. 1. (01 May 2000), pp. 25-29, doi:10.1038/75556.

[3] Controlling the False Discovery Rate: A Practical and Powerful Approach to Multiple Testing. Journalof the Royal Statistical Society. Series B (Methodological), Vol. 57, No. 1. (1995), pp. 289-300,doi:10.2307/2346101.

**7. KEGG pathway Enrichment Analysis**

Biological Pathway analysis is based on the Kyoto encyclopedia of genes and genomes (KEGG) biological pathway database (http://www.genome.jp/). This analysis starts from the perspective of complex regulatory networks and conducts biological pathways on gene collections Enrichment analysis is an important method for studying biological functions. We put the DEGs into biological pathways for comprehensive analysis, and analyze the degree and law of the influence of functional variations on biological pathways, so as to provide help for subsequent verification and functional experiments.

Gene function enrichment analysis is mainly divided into two steps:

**Frist, Gene KEGG biological pathway annotation:**

KEGG pathway annotation provides information on signal transduction and disease pathway annotations for DEGs, thereby providing background knowledge of gene pathways and functional research.

**Second, Enrichment analysis:**

Calculate the P value by Fisher Exact Test, and use P<0.05 as the significance threshold to obtain statistically significant signal transduction and disease pathways relative to the background, so as to obtain the distribution information and significance of the gene set in the KEGG category.

**References:**

[1] KEGG: Kyoto Encyclopedia of Genes and Genomes. Nucleic Acids Research, Vol. 28, No. 1. (01 January 2000), pp. 27-30, doi:10.1093/nar/28.1.27

[2] A direct approach to false discovery rates. Journal of the Royal Statistical Society: Series B (Statistical Methodology), Vol. 64, No. 3. (August 2002), pp.479-498, doi:10.1111/1467-9868.00346
