# Supplementary figures and images for: The Expression Profiles of mRNAs and lncRNAs in Buffalo Muscle Stem Cells Driving Myogenic Differentiation
Source: Front Genet. 2021 Jul 7;12:643497. doi: 10.3389/fgene.2021.643497 (PMC8294193; doi:10.3389/fgene.2021.643497)

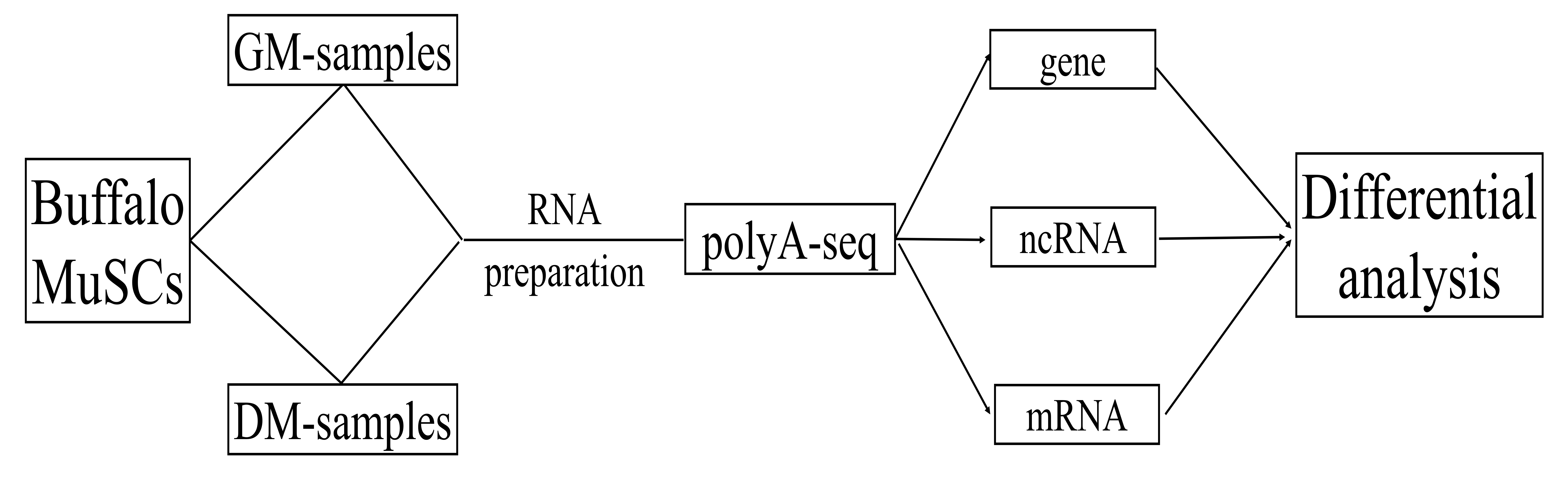

Supplement: Supplementary file 10 [file Image_1.TIF]

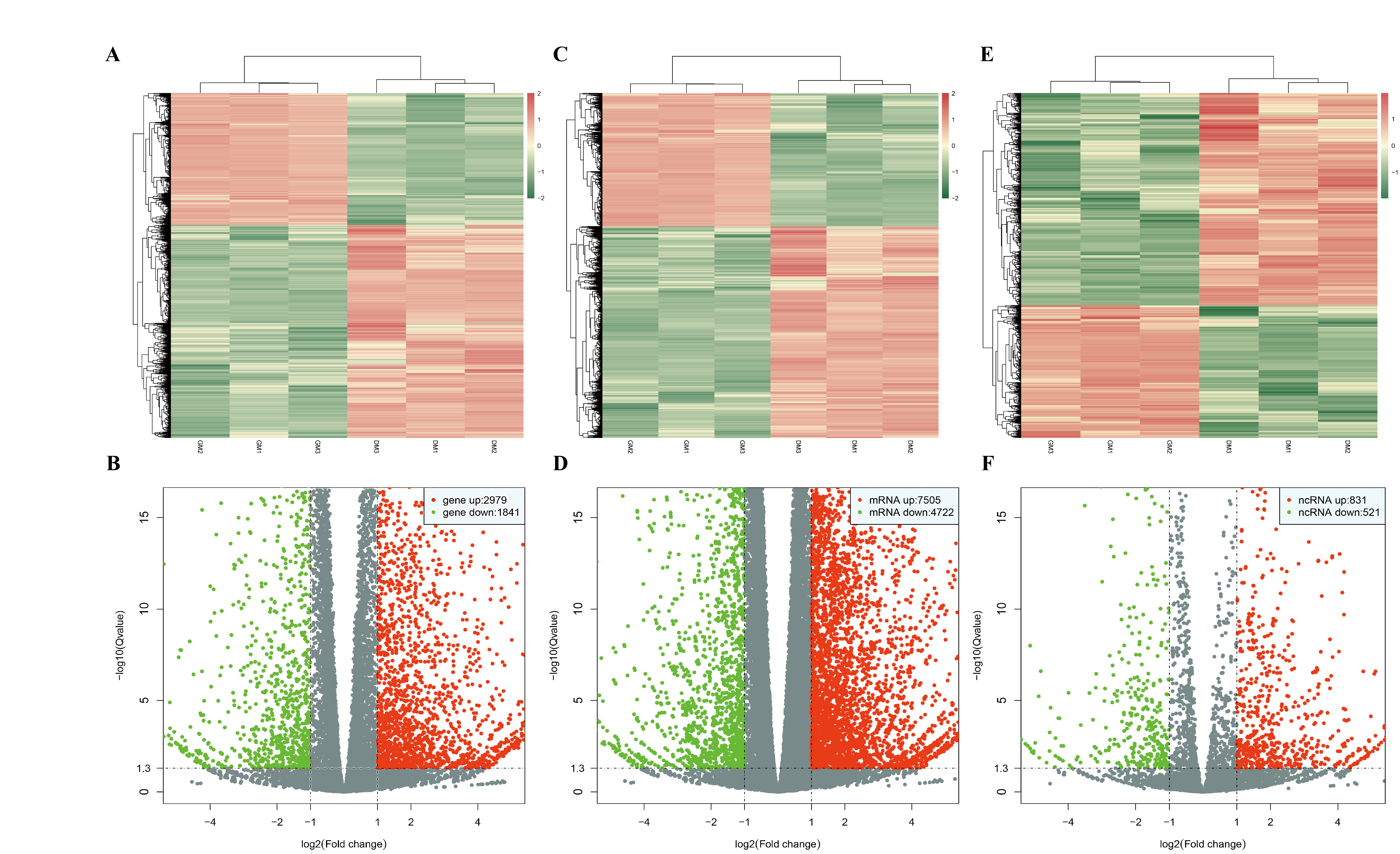

Supplement: Supplementary file 11 [file Image_2.TIF]

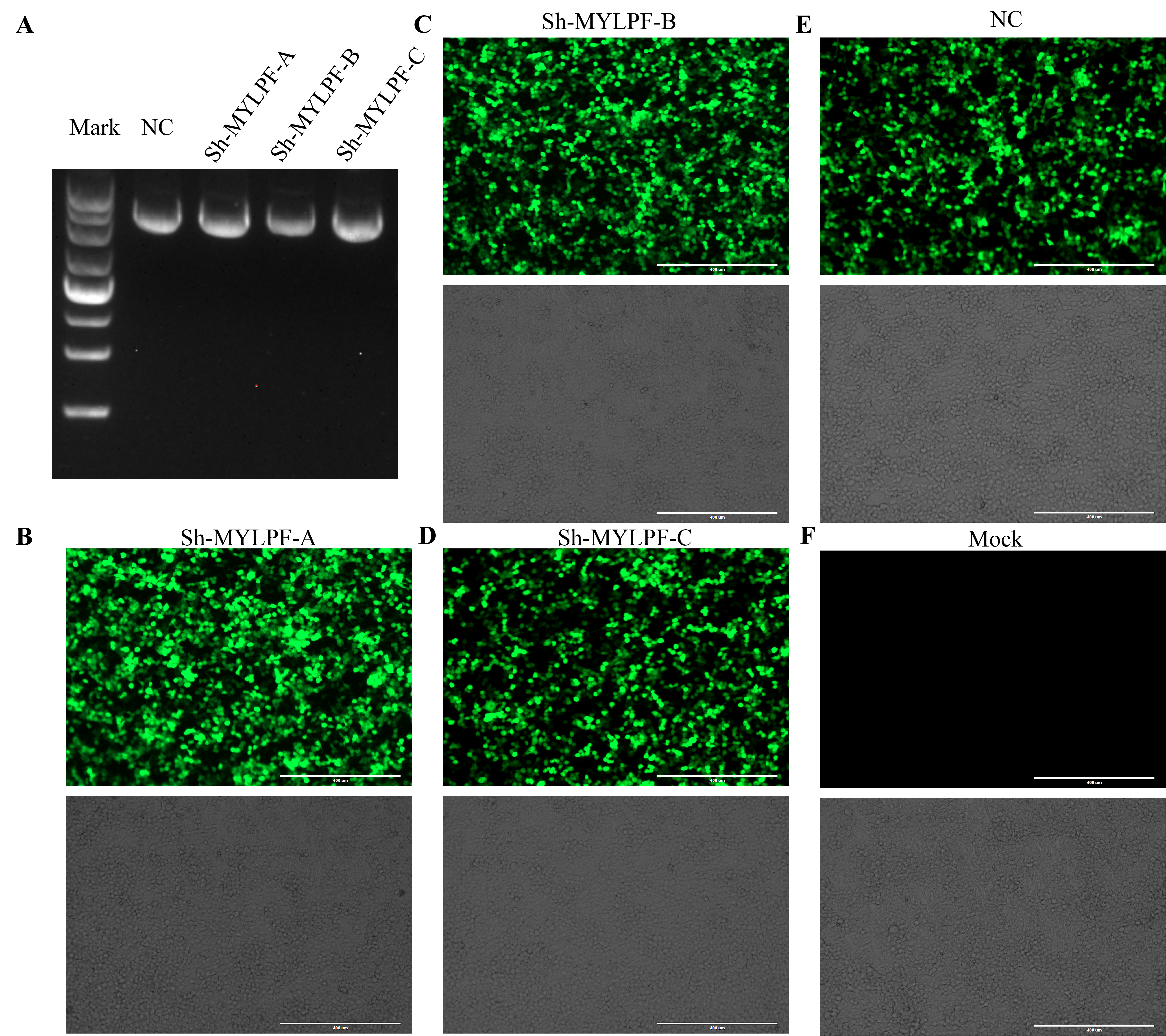

Supplement: Supplementary file 12 [file Image_3.JPEG]

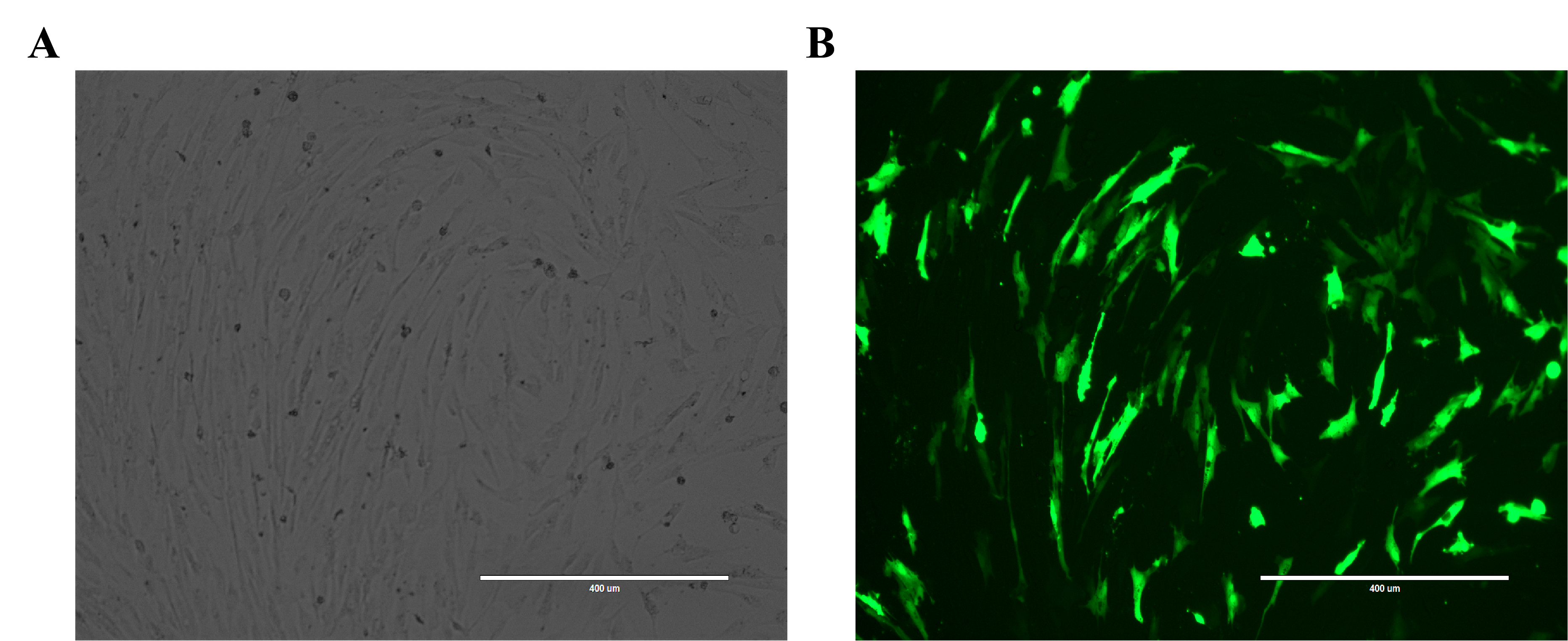

Supplement: Supplementary file 13 [file Image_4.TIF]
